# Development of the Japanese Anticholinergic Risk Scale: English translation of the Japanese article

**DOI:** 10.1111/ggi.15001

**Published:** 2024-12-05

**Authors:** Fumihiro Mizokami, Tomohiro Mizuno, Rena Taguchi, Izumi Nasu, Sayaka Arai, Keiichiro Higashi, Ayaka Matsumoto, Miwako Kamei, Taro Kojima, Takayoshi Sakai, Yuuka Shibata, Yasushi Takeya, Masaki Mogi, Shizuo Yamada, Masahiro Akishita, Japanese Society of Geriatric Pharmacy Working Group on Japanese Anticholinergic Risk Scale

**Affiliations:** ^1^ Department of Pharmacy National Center for Geriatrics and Gerontology Obu Japan; ^2^ Department of Pharmacotherapeutics and Informatics Fujita Health University School of Medicine Toyoake Japan; ^3^ Research Department, Institute for Health Economics and Policy Association for Health Economics Research and Social Insurance and Welfare Tokyo Japan; ^4^ Flatiron Health K.K. Tokyo Japan; ^5^ Division of Pharmacy Chiba University Hospital Chiba Japan; ^6^ Department of Pharmacy Asanogawa General Hospital Kanazawa Japan; ^7^ Center for Sarcopenia and Malnutrition Research Kumamoto Rehabilitation Hospital Kumamoto Japan; ^8^ Faculty of Pharmaceutical Sciences Teikyo Heisei University Tokyo Japan; ^9^ Department of Geriatric Medicine The University of Tokyo Tokyo Japan; ^10^ Department of Rehabilitation for Orofacial Disorders Osaka University Graduate School of Dentistry Osaka Japan; ^11^ Department of Pharmaceutical Services Hiroshima University Hospital Hiroshima Japan; ^12^ Department of Geriatric Nursing Osaka University Graduate School of Medicine Osaka Japan; ^13^ Department of Pharmacology Ehime University Graduate School of Medicine Matsuyama Japan; ^14^ Center for Pharma‐Food Research (CPFR), Graduate School of Pharmaceutical Sciences University of Shizuoka Shizuoka Japan; ^15^ Tokyo Metropolitan Institute for Geriatrics and Gerontology Tokyo Japan

**Keywords:** adverse drug reactions, anticholinergic risk scale, Delphi method

## Abstract

**Background:**

Anticholinergic burden, reflecting the cumulative impact of medications with anticholinergic properties, significantly predicts adverse drug reactions and geriatric syndromes in older adults. Although anticholinergic risk scales (ARS) have been developed and validated in various countries, none have been tailored specifically for Japan. The Japanese Anticholinergic Risk Scale (JARS) was developed to adapt the existing ARS frameworks to the Japanese context, considering unique medication profiles and cultural factors.

**Process:**

First, a systematic review was performed to follow the protocol registered in PROSPERO (CRD42017076510). A PubMed search from October 2017 to March 2023 was conducted to identify ARS publications post‐September 2017. Based on two algorithms, average scores from the existing scores were used to develop JARS. The Delphi method, an expert consensus approach, was applied to determine the scores for medications that were not established by the algorithms. Sixteen articles identified in our systematic review contributed to JARS development. JARS categorizes 158 medications into three potency groups: 37 drugs scored as 3 (strong), 27 as 2 (moderate), and 94 as 1 (weak).

**Conclusion:**

JARS, the newly developed ARS, could be a critical tool for anticholinergic burden assessment in older Japanese populations. Developed through a systematic review and Delphi‐based expert consensus, it encompasses 158 medications, offering a comprehensive anticholinergic burden assessment. Future studies and updates should be conducted to improve the accuracy and clinical applicability of this scale. **Geriatr Gerontol Int 2025; 25: 5–13**.

## Background

Polypharmacy, defined as the concurrent use of multiple medications, significantly increases the risk of adverse drug reactions, drug–drug interactions, and specific geriatric syndromes such as falls, delirium, and cognitive impairment.^1^ In Japan, the challenge of polypharmacy is exacerbated by an aging population and a high prevalence of chronic diseases.^2^, ^3^ Adverse drug‐related problems associated with polypharmacy among older adults pose a serious concern for global healthcare.^4^, ^5^, ^6^

Anticholinergic medications are commonly prescribed to manage various conditions in older adults, including overactive bladder, depression, and Parkinson's disease.^7^ However, these medications inhibit the activity of acetylcholine, a neurotransmitter crucial for cognitive function and other physiological processes.^8^ The anticholinergic burden (ACB), reflecting the cumulative impact of medications with anticholinergic properties, is a significant predictor of adverse drug reactions in older adults.^9^, ^10^ Elevated ACB levels correlate with increased risks of cognitive impairment, falls, functional decline, and mortality in this population.^11^, ^12^

Several anticholinergic risk scales (ARS) have been developed to quantify ACB and assess the potential risk for adverse anticholinergic drug events.^13^ These scales assign medication scores based on their anticholinergic potency, providing an overall assessment of the anticholinergic properties of the prescribed medications.^14^ Incorporating ARS into clinical practice has proven effective in optimizing medication regimens and enhancing medication safety for older adults.^15^

Although ARS have been validated in countries outside Japan,^14^, ^16^, ^17^ their applicability to the Japanese population may be limited due to differences in medication availability, prescribing practices, and cultural factors. Research conducted in Japan has revealed age‐related increases in anticholinergic drug use, although the contribution of different drug types to total ACB varies across scales. For example, the number of anticholinergic medications increases with age according to the ACB scale and Anticholinergic Drug Scale but decreases when assessed using the ARS and Beers criteria.^18^ Studies have linked anticholinergic drug use, particularly polypharmacy and cumulative sedative or anticholinergic doses, with an elevated risk of long‐term care needs among patients with cognitive impairment.^19^ Discontinuing these medications in patients with dementia has been shown to reduce adverse drug reactions.^20^ These studies underscore the importance of using a Japan‐specific scale to accurately gauge the ACB in this demographic.

In this report, we aimed to develop the Japanese Anticholinergic Risk Scale (JARS) by systematically reviewing existing ARS, refining their scoring criteria through expert consensus, and incorporating medications authorized in Japan to comprehensively assess ACB in older adults. Developing and validating the JARS is essential for accurately assessing ACB and guiding clinical decisions tailored to the Japanese population. JARS aims to adapt existing ARS frameworks to the unique medication profiles and cultural considerations in Japan, filling a critical gap in ACB assessment and promoting enhanced medication safety and personalized care for older adults in Japan.

## Development of the Japanese Anticholinergic Risk Scale

### Literature screening

**Figure 1:**
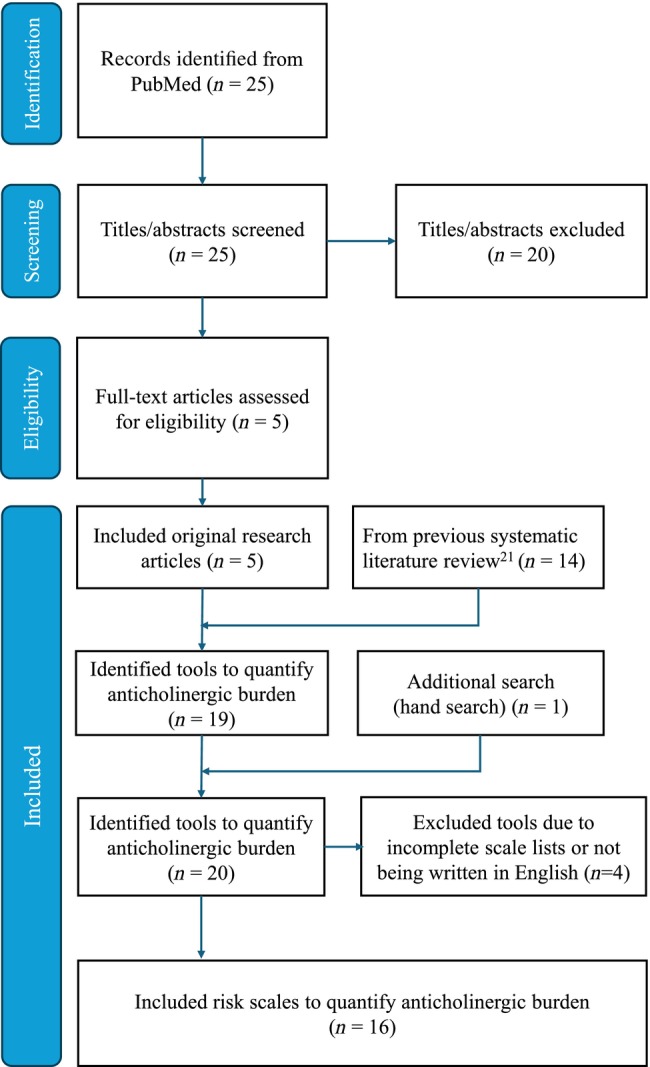
PRISMA flow diagram of literature search and selection process for developing the Japanese Anticholinergic Risk Scale. The process began with the identification of 25 articles in PubMed. Titles and abstracts of these records were screened, leading to the exclusion of 20 records. Five original research articles were included and assessed for eligibility, along with 14 articles from a previous systematic literature review. Additionally, one tool was manually identified through a hand search. After excluding four tools due to incomplete scale lists or not being written in English, 16 articles were included in the final analysis.

Hanlon *et al*. investigated the risks associated with polypharmacy involving medications with anticholinergic properties and identified 14 relevant ARS published up to September 2017.^21^ Our systematic review followed their methods registered in PROSPERO (CRD42017076510).^21^ To identify ARS published after September 2017, we extended our search in PubMed from October 2017 to March 2023 using the search terms “anticholinergic [Title/Abstract] AND burden [Title/Abstract] AND (scale [Title/Abstract] OR list [Title/Abstract] OR score [Title/Abstract] OR tool [Title/Abstract]) AND review.” Articles were included if they developed scales quantifying ACB or original research papers presenting ARS in English. The initial literature review identified 25 articles, with six additional articles from Hanlon *et al*.^21^ After a full‐text review, four articles were excluded due to incomplete scale lists or non‐English language. The remaining 16 articles^11^, ^12^, ^14^, ^16^, ^17^, ^22^, ^23^, ^24^, ^25^, ^26^, ^27^, ^28^, ^29^, ^30^, ^31^, ^32^ formed the basis for developing the JARS (Figure 1).

All 16 scales from the selected articles were included for developing the JARS as they met the following criteria in that they: (1) provided drug lists with grading scores for quantifying ACB; (2) were based on existing pharmacological activity assessments, literature reviews, and clinical expert opinions; and (3) provided lists comparable with other scales. None of the scales were excluded based on these criteria. Details of the existing ARS are provided in Table 1. The existing ARS were classified into two groups based on the methodology used for their development: those developed through a combination of pharmacological activity assessments, literature reviews, and expert opinions (evaluation method 1), and those based solely on pharmacological measurements of cholinergic activity (evaluation method 2). These studies collectively listed 286 drugs and scored them from 0 (no anticholinergic activity) to 3 or 4 (high anticholinergic potency). Drugs not authorized in Japan or intended for systemic effects were excluded, resulting in 185 drugs evaluated using JARS. These scales varied in the number of medications included (ranging from 22 to 151), disease specificity (some focusing on conditions such as dementia or Parkinson disease), and the methodology used for development (most relying on pharmacological activity assessment, literature review, and expert opinion).

**Table 1:** List of anticholinergic risk scales

Scale	Publish year	Evaluation method	Number of drugs (excluding score of 0)	Specificity
ADS^22^	2006	1	127	Residents of care facilities
ABC^23^	2006	1	27	None
ACoB^24^	2008	1	88	Dementia
CrAS^11^	2008	1	60	≥65 years
ARS^12^	2008	1	49	≥65 years
AAS^25^	2010	1	99	Parkinson disease
ALS^26^	2011	1	49	Dementia
mARS^27^	2014	1	61	None
AIS^28^	2017	1	128	Mental disorders
AEC^29^	2017	1	60	Dementia
ACB^14^	2018	1	151	None
BAAS^30^	2019	1	125	None
KABS^17^	2019	1	138	None
Swe‐ABS^16^	2023	1	104	None
Chew‐AAS^31^	2008	2	22	None
Yamada‐ABS^32^	2023	2	96	None

Evaluation Method 1: Developed through a combination of existing pharmacological activity assessments, literature reviews, and expert opinions.

Evaluation Method 2: Developed solely based on pharmacological cholinergic activity measurements.

AAS, Anticholinergic Activity Scale; ABC, Anticholinergic Burden Classification; ACB, anticholinergic burden; ACoB, Anticholinergic Cognitive Burden; ADS, Anticholinergic Drug Scale; AEC, anticholinergic effect on cognition; AIS, anticholinergic impregnation scale; ALS, Anticholinergic Loading Scale; ARS, Anticholinergic Risk Scale; BAAS, Brazilian Anticholinergic Activity Scale; Chew‐AAS, Chew‐Anticholinergic Activity Scale; CrAS, Clinician‐rated Anticholinergic Scale; KABS, Korean Anticholinergic Burden Scale; mARS, modified Anticholinergic Risk Scale; Swe‐ABS, Sweden Anticholinergic Burden Scale; Yamada‐ABS, Yamada‐Anticholinergic Burden Scale.

### Process of scoring

Scores from the 16 articles were standardized for consistency. Scores of 0 were removed from the existing scales due to insufficient pharmacological evaluation, and scores of 4 on the Anticholinergic Activity Scale were adjusted to 3.^25^ Therefore, only drugs with a score of 1 or higher on the existing scales were included in the development of JARS.

The average score was calculated from these articles, and the JARS score was determined using the following algorithm:

- If a drug received scores from two or more existing scales with complete agreement, that score was assigned (type 1).
- If scores differed by 1 point among two or more scales, an average score was calculated. Scores of ≤0.3 were rounded down, and scores of ≥0.7 were rounded up to determine the final score (type 2).
- Drugs with score discrepancies of 2 points or more among existing scales, or scoring 1–3 on only one scale, had their scores determined by expert consensus using the Delphi method (type 3).

A panel of six experts, comprising geriatricians (TK, MM, MA) and geriatric pharmacists (FM, TM, RT), with MM and TM also serving as geriatric pharmacologists, convened to assign medication scores classified as type 3. The experts evaluated each medication based on its mechanism of action, adverse drug reactions documented in Japanese package inserts, and relevant literature. Each expert independently assigned a score to each drug using a scale from 1 to 3. The panel then convened for discussions and repeated the scoring process and discussion twice. A consensus was defined as ≥80% agreement among the experts. If a consensus was not reached after two rounds of scoring, the drug was excluded from the final version of JARS.

Among the 185 drugs evaluated, the type 1 algorithm was applied to 107 drugs to determine their scores. For the remaining 78 drugs, the type 2 algorithm was used to assign scores to 12 drugs, while the scores of the other 66 drugs were determined through expert consensus using the Delphi method. Using the Delphi method (algorithm type 3), consensus was reached for 39 drugs, whereas 27 drugs did not achieve consensus and were consequently excluded. These exclusions were primarily due to factors such as being evaluated by only one existing scale or insufficient evidence of anticholinergic effects documented in Japanese drug package inserts. The included drugs were categorized based on their anticholinergic potency as follows:

- Score 3 (strong anticholinergic effect): 37 drugs, including 15 over‐the‐counter (OTC) drugs (40.5%).
- Score 2 (moderate anticholinergic effect): 27 drugs, including 4 OTC drugs (14.8%).
- Score 1 (weak anticholinergic effect): 94 drugs, including 17 OTC drugs (19.1%).

Specific scores for each drug are detailed in Table 2.

**Table 2:** Medication lists included in the Japanese Anticholinergic Risk Scale

Drug class	Medication	ATC code	Score	OTC drugs
Benzodiazepines	Triazolam	N05CD05	1	
	Estazolam	N05CD04	1	
	Flunitrazepam	N05CD03	1	
	Flurazepam	N05CD01	1	
	Alprazolam	N05BA12	1	
	Clorazepate	N05BA05	1	
	Chlordiazepoxide	N05BA02	1	
	Lorazepam	N05BA06	1	
	Diazepam	N05BA01	1	
	Clonazepam	N03AE01	1	
Antiepileptic drugs	Phenobarbital	N03AA02	1	
	Carbamazepine	N03AF01	2	
	Valproic acid	N03AG01	1	
Parkinson disease treatment drugs	Carbidopa ± levodopa	N04BA02	1	
	Levodopa	N04BA01	1	
	Amantadine	N04BB01	2	
	Pramipexole	N04BC05	1	
	Bromocriptine	G02CB01	1	
	Rotigotine	N04BC09	1	
	Trihexyphenidyl/benzhexol	N04AA01	3	
	Biperiden	N04AA02	3	
	Selegiline	N04BD01	1	
	Entacapone	N04BX02	1	
Phenothiazine antipsychotics	Chlorpromazine	N05AA01	3	
	Prochlorperazine	N05AB04	2	
	Propericiazine/periciazine	N05AC01	2	
	Fluphenazine	N05AB02	2	
	Perphenazine	N05AB03	2	
	Levomepromazine/methotrimeprazine	N05AA02	2	
Butyrophenone antipsychotics	Haloperidol	N05AD01	1	
Multi‐acting receptor‐targeted antipsychotics	Clozapine	N05AH02	3	
	Olanzapine	N05AH03	2	
	Quetiapine	N05AH04	2	
	Asenapine	N05AH05	1	
Serotonin–dopamine antagonists	Paliperidone	N05AX13	1	
	Blonanserin	0	1	
	Risperidone	N05AX08	1	
Dopamine D2 receptor partial agonists	Aripiprazole	N05AX12	1	
Other antipsychotics	Zotepine	N05AX11	2	
Mood stabilizers	Lithium	N05AN01	1	
Serotonin–norepinephrine reuptake inhibitors	Duloxetine	N06AX21	1	
	Venlafaxine	N06AX16	1	
	Paroxetine	N06AB05	2	
	Escitalopram	N06AB10	1	
	Sertraline	N06AB06	1	
	Fluvoxamine	N06AB08	1	
Tricyclic antidepressants	Amitriptyline	N06AA09	3	
	Amoxapine	N06AA17	3	
	Imipramine	N06AA02	3	
	Clomipramine	N06AA04	3	
	Trimipramine	N06AA06	3	
	Nortriptyline	N06AA10	3	
	Dothiepin/dosulepin	N06AA16	2	
	Lofepramine	N06AA07	2	
Tetracyclic antidepressants	Setiptiline	0	2	
	Maprotiline	N06AA21	2	
	Mianserin	N06AX03	2	
Noradrenergic and specific serotonergic antidepressants	Mirtazapine	N06AX11	1	
Other antidepressants	Trazodone	N06AX05	1	
Centrally acting muscle relaxants	Tizanidine	M03BX02	3	
	Eperisone	M03BX09	2	
	Chlorzoxazone	M03BB03	2	○
	Baclofen	M03BX01	2	
	Methocarbamol	M03BA03	1	●
Antiemetics and antivertigo drugs	Difenidol	0	3	
Central antiemetics and antivertigo drugs	Dimenhydrinate	R06AA11	3	
Muscarinic cholinergic receptor antagonists	Hyoscine/scopolamine	A04AD01	3	○
Triptans (5‐HT1B/1D receptor agonists)	Sumatriptan	N02CC01	1	
	Zolmitriptan	N02CC03	1	
	Naratriptan	N02CC02	1	
Digitalis preparations	Digoxin	C01AA05	1	
Nitrates	Isosorbide mononitrate	C01DA14	1	
	Isosorbide dinitrate	C01DA08	1	
Other coronary vasodilators	Dipyridamole	B01AC07	1	
Na channel blockers (Class Ia)	Disopyramide	C01BA03	2	
	Quinidine	C01BA01	1	
Class III antiarrhythmics	Amiodarone	C01BD01	1	
Angiotensin‐converting enzyme inhibitors	Captopril	C09AA01	1	
	Trandolapril	C09AA10	1	
	Benazepril	C09AA07	1	
Calcium channel blockers	Diltiazem	C05AE03	1	
	Nifedipine	C08CA05	1	
Beta blockers	Atenolol	C07AB03	1	
	Betaxolol	C07AB05	1	
	Metoprolol	C07AB02	1	
Vasodilators	Hydralazine	C02DB02	1	
Osmotic diuretics	Isosorbide	0	1	
Potassium‐sparing diuretics	Triamterene	C03DB02	1	
Loop diuretics	Furosemide	C03CA01	1	
Centrally acting non‐narcotic antitussives	Cloperastine	R05DB21	2	
	Dextromethorphan	R05DA09	1	●
	Codeine	R05DA04	1	●
Cough expectorants	Guaifenesin	R05CA03	1	●
Xanthine derivatives	Theophylline	R03DA04	2	●
Aggressive factor inhibitors	Atropine	A03BA01	3	
	Tiquizium	0	3	●
	Butylscopolamine	A03BB01	3	●
	Propantheline	A03AB05	3	
	Belladonna	A03BA04	3	●
Intestinal motility inhibitors	Loperamide	A07DA03	1	
Histamine (H2) receptor antagonists	Cimetidine	A02BA01	2	●
	Nizatidine	A02BA04	1	●
	Famotidine	A02BA03	1	
Proton pump inhibitors	Lansoprazole	A02BC03	1	●
Protective factor combinations	Dicycloverine/dicyclomine	A03AA07	3	●
Opioid agonists	Trimebutine	A03AA05	1	
Dopamine receptor antagonists	Domperidone	A03FA03	1	
	Metoclopramide/Reglan	A03FA01	1	●
Adrenal corticosteroids	Cortisone	A01AC03	1	●
	Dexamethasone	A01AC02	1	●
	Triamcinolone	A01AC01	1	●
	Hydrocortisone	A01AC03	1	●
	Prednisolone	A01AC04	1	
	Methylprednisolone	D07AA01	1	
Selective muscarinic receptor antagonists	Imidafenacin	G04BD14	3	
	Solifenacin	G04BD08	3	
	Tolterodine	G04BD07	3	
	Fesoterodine	G04BD11	3	
Anticholinergic + calcium channel blocking agents	Oxybutynin	G04BD04	3	●
	Propiverine	G04BD06	3	●
Other overactive bladder treatments	Flavoxate	G04BD02	3	
Coumarin derivatives	Warfarin	B01AA03	1	
Gout attack remission and prevention drugs	Colchicine	M04AC01	1	
Biguanides	Metformin	A10BA02	1	
Immunosuppressants	Azathioprine	L04AX01	1	
	Cyclosporine	L04AD01	1	
	Methotrexate	L01BA01	1	○
Histamine (H1) receptor antagonists (first generation)	Carbinoxamine	R06AA08	3	●
	Clemastine	D04AA14	3	●
	Chlorpheniramine	R06AB04	3	○
	Diphenylpyraline	R06AA07	3	●
	Diphenhydramine	D04AA32	3	
	Cyproheptadine	R06AX02	3	
	Hydroxyzine	N05BB01	3	○
	Pheniramine	D04AA16	3	●
	Promethazine	D04AA10	3	●
	Alimemazine	R06AD01	2	●
Histamine (H1) receptor antagonists (second generation)	Mequitazine	R06AD07	3	●
	Cetirizine	R06AE07	2	●
	Epinastine	R06AX24	1	
	Emedastine	S01GX06	1	
	Olopatadine	R01AC08	1	●
	Ketotifen	R06AX17	1	
	Desloratadine	R06AX27	1	●
	Fexofenadine	R06AX26	1	
	Rupatadine	R06AX28	1	
	Levocetirizine	R06AE09	1	●
	Loratadine	R06AX13	1	○
Glycopeptide antibiotics	Vancomycin	A07AA09	1	
Lincosamide antibiotics	Clindamycin	D10AF01	1	
Broad‐spectrum penicillins	Ampicillin	J01CA01	1	
Non‐steroidal anti‐inflammatory drugs	Celecoxib	L01XX33	1	
Other opioids	Tramadol	N02AX02	2	
Morphinan opioids	Oxycodone	N02AA05	1	
	Morphine	N02AA01	1	
Phenylpiperidine opioids	Fentanyl	N01AH01	1	
Other opioids	Methadone	N07BC02	2	
	Tapentadol	N02AX06	1	

○, over‐the‐counter (OTC) drugs only; ●, both OTC and prescription drugs. Blank indicates prescription drugs only in Japan.

#### How to use the Japanese Anticholinergic Risk Scale

The JARS is primarily designed for use among older adults; however, it is not limited to this demographic. Young individuals with underlying conditions that predispose them to adverse drug reactions can also benefit from the application of JARS. The process of JARS use is simpler than that employed for other ARSs.^14^, ^24^ JARS is intended for use in various healthcare settings and can be utilized by healthcare professionals, including pharmacists, physicians, dentists, nurses, and others. JARS recommends evaluating ACB from two aspects:

1. Individual Drug Risk Assessment: JARS assigns a score from 1 to 3 to each drug based on its anticholinergic properties. Healthcare professionals should consider switching from a medication with a higher score to the one with a lower score.
2. Comprehensive Risk Assessment: Older adults take multiple medications due to various comorbidities. Healthcare professionals can determine a patient's total ACB by adding the individual drug scores.

#### Primary characteristics of the Japanese Anticholinergic Risk Scale

JARS, which exclusively includes drugs authorized in Japan, serves as a comprehensive tool for assessing ACB in the Japanese population, designed for public use. It encompasses 158 medications across various drug classes commonly prescribed in Japan. JARS aligns with the trends observed in other ARS, such as ACB,^14^ the Brazilian Anticholinergic Activity Scale,^30^ the Korean Anticholinergic Burden Scale (KABS),^17^ and Swe‐ABS,^16^ which cover a wide array of medications targeting diverse diseases and patient populations. Before 2017, many ARS focused on specific diseases or patient groups, leading to varying scopes of application among different scales. Some scales may not adequately evaluate the risk posed by OTC drugs in the general population. OTC drugs are widely used across all age groups and are easy to purchase. However, there is limited documentation regarding the inclusion of OTC drugs in each ARS within specific countries or regions. In JARS, >40% of OTC drugs were categorized with a score of 3. OTC drugs are readily accessible at pharmacies in Japan and other countries, making it crucial for JARS to delineate drugs that are available for public purchase. This feature of JARS aims to raise awareness among patients and pharmacy staff about the risks associated with anticholinergic OTC drugs.

#### Comparison with other anticholinergic risk scales

JARS also incorporates the Yamada‐Anticholinergic Burden Scale (Yamada‐ABS),^32^ introduced in 2023. Although developed based on pharmacological assessments of cholinergic activity, Yamada‐ABS^32^ evaluated the anticholinergic effects of frequently prescribed drugs (260 drugs) in Japan. Earlier ARS, such as those released in 2008^12^ and the modified ARS,^27^ utilized databases from the National Institute of Mental Health psychoactive drug screening program and the British National Formulary, respectively, which predominantly feature drugs with known anticholinergic activity. However, no such database exists in Japan, despite the widespread use of such medications. Yamada‐ABS^32^ lists 96 drugs, evaluating a higher number of medications compared with the Chew‐Anticholinergic Activity Scale,^31^ which is also based on pharmacological anticholinergic activity. Thus, JARS potentially bridges existing gaps in the ACB assessment specific to Japan. To enhance the reliability of JARS, the development of a national database focusing on anticholinergic activity within Japan is recommended.

#### Methodological strengths

JARS was developed using a methodology that included a systematic literature review and expert consensus through the Delphi method. This approach aimed to enhance the validity and reliability of the scale by minimizing individual biases and fostering consensus among experts. However, JARS differs in its drug‐scoring methodology compared with major existing ARS^12^ such as ACB^14^ released in 2018, KABS,^17^ and Swe‐ABS,^16^ which utilize a 0–3 scoring system. These scales were developed based on existing pharmacological activity assessments, literature reviews, and expert opinions, with scores determined by these ARS^12^ and expert consensus. Yamada‐ABS^32^ also used a 0–3 scoring system based on muscarinic receptor binding activities (IC_50_). However, each scale varies in how they assign a score of 0, often due to limited evidence supporting the absence of anticholinergic effects for each drug in Japan. Despite deliberation by a panel of six experts, no consensus was reached on an ideal weighted scoring method for JARS, resulting in the scale's decision not to adopt a score of 0.

#### Limitations of the Japanese Anticholinergic Risk Scale

JARS acknowledges several limitations that warrant consideration. First, its coverage is restricted to oral medications and transdermal patches with systemic effects, excluding topical and inhaled medications with localized effects or variable systemic absorption. ACB,^14^ released in 2018, incorporates inhalation drugs, which are likely to exhibit systemic effects. Therefore, future revisions of JARS should encompass these medications, particularly inhaled drugs, given their potential systemic impact influenced by inhalation techniques contributing to ACB. Secondly, JARS employs a three‐point ordinal scoring system^1^, ^2^, ^3^ to categorize medications based on their anticholinergic potency. However, this system may not linearly correspond to the degree of anticholinergic effect; a drug with a score of 2 may not precisely indicate twice the anticholinergic potency of a drug with a score of 1. To mitigate overall ACB in prescriptions, consideration should be given to switching to alternative drugs with lower ACB scores.^14^, ^24^ Thirdly, JARS does not accommodate variations in dosage or individual pharmacokinetics, crucial factors influencing the ACB on patients, particularly in older adults with altered drug metabolism and clearance due to age‐related organ function changes.^33^ In addition, the duration of anticholinergic drug use is another factor that JARS currently does not account for in its scoring system. However, longer‐term use of anticholinergic medications, even those with lower JARS scores, may increase the risk of adverse effects such as cognitive impairment or physical decline.^9^, ^10^ This limitation highlights the importance of considering both dosage and duration when evaluating the ACB. Fourth, JARS does not assess the interaction between other medications not included in JARS and anticholinergic drugs. However, geriatricians and geriatric pharmacists should actively monitor for anticholinergic side effects, such as dry mouth, constipation, or confusion, which may be induced by the concomitant use of these drugs. Furthermore, JARS does not encompass all medications available in Japan, such as herbal medicines and newly approved drugs. Some drugs with potential anticholinergic effects might not be included due to insufficient data or lack of expert consensus. Therefore, ongoing evaluations and updates of JARS are essential to ensure its comprehensive coverage and relevance amid Japan's evolving medication landscape.

#### Overview and future research directions

JARS serves as a valuable tool for assessing ACB in older Japanese adults. Developed through a systematic literature review and expert consensus using the Delphi method, JARS incorporates 158 medications, offering a comprehensive assessment of ACB. The inclusion of both prescribed medications and OTC drugs enhances its relevance to general clinical practice and public awareness.

However, future research should address its limitations to enhance accuracy and clinical utility. For instance, integrating pharmacokinetic data and broadening its scope to include inhaled medications and topical treatments with potential systemic effects could enhance its applicability. Continuous updates to JARS are essential to align with Japan's dynamic pharmaceutical landscape.

## Funding information

All expenses related to the development of the JARS were funded by the Japanese Society of Geriatric Pharmacy, with no additional external funding.

## Disclosure statement

Regarding conflict of interest (COI), all 15 members reported the state of COI with respect to their economic relationship with companies involved in geriatric pharmacology according to the COI detailed regulations of the Japanese Society of Geriatric Pharmacy, which were prepared based on the “Common Guidelines regarding the Conflict of Interest in Clinical Studies” established by the Japanese Society of Internal Medicine and affiliated societies: Companies/corporations from which the members or their relatives in the first degree, as a person, obtained rewards: Executive rewards (1&amp;#x02009;000&amp;#x02009;000 yen or more), shares (1&amp;#x02009;000&amp;#x02009;000 yen or more, or 5% or more of the stock), patent fee (1&amp;#x02009;000&amp;#x02009;000 yen or more), lecture/manuscript fee (500&amp;#x02009;000 yen or more), research funds/grants (1&amp;#x02009;000&amp;#x02009;000 yen or more), travel expenses/gifts (50&amp;#x02009;000 yen or more). Companies/corporations responsible for cooperative industrial‐academic activities with departments to which the members belong. Scholarship funds (2&amp;#x02009;000&amp;#x02009;000 yen or more), belonging to contribution lectures sponsored by companies. The COI committee of the Japanese Society of Geriatric Pharmacy reviewed the COI checklists provided by all authors before the establishment. If there were any kinds of personal conflict with the content of the guidelines, they were investigated by the committee whether there was any influence on the guidelines and consequently confirmed that there were no problems within the declaration of the COI. As a method to open COI in the guidelines, the names of companies reported by members responsible for preparation are presented below in reference to the guidelines prepared by other societies. The names of companies reported are as follows (inspection period: from January 1, 2021 to December 31, 2023). Their names are expressed as those as of May 2024 (in alphabetical order). However, neither publishing companies nor corporations taking a neutral stand are included. Companies/corporations from which the members or their relatives in the first degree, as a person, obtained rewards: Astellas Pharma Inc., Bayer Yakuhin, Ltd., Daiichi Sankyo Company, Limited, Eisai Co., Ltd., EM Systems Co., Ltd., Fukuda Denshi Co., Ltd., Kracie Pharma, Ltd., Meiji Seika Pharma Co., Ltd., Mitsubishi Tanabe Pharma Corporation, Pfizer Inc., Toa Eiyo Ltd., Towa Pharmaceutical Co., Ltd., and Tsumura &amp; Co. Company/corporation responsible for cooperative industrial‐academic activities with departments to which the members belong: Sugi Pharmacy Co., Ltd.

## Author contributions

Fumihiro Mizokami conceptualized the study with support from Tomohiro Mizuno, Rena Taguchi and Masahiro Akishita. Fumihiro Mizokami, Tomohiro Mizuno and Rena Taguchi conducted the systematic review. Fumihiro Mizokami and Tomohiro Mizuno drafted the original manuscript. All authors (Fumihiro Mizokami, Tomohiro Mizuno, Rena Taguchi, Izumi Nasu, Sayaka Arai, Keiichiro Higashi, Ayaka Matsumoto, Miwako Kamei, Taro Kojima, Takayoshi Sakai, Yuuka Shibata, Yasushi Takeya, Masaki Mogi, Shizuo Yamada, and Masahiro Akishita) participated in the assessment of drugs and made contributions to the manuscript and its conclusions. All authors reviewed the final manuscript.

## Data Availability

The data supporting the findings of this study are available from the corresponding author, Fumihiro Mizokami, upon request.
